# Supplementary material for: Surface Hydration Protects Cystic Fibrosis Airways from Infection by Restoring Junctional Networks
Source: Cells. 2022 May 9;11(9):1587. doi: 10.3390/cells11091587 (PMC9105190; doi:10.3390/cells11091587)
Supplement: Supplementary file 1 [file cells-11-01587-s001.zip › cells-1663712 Supplementary.pdf]

cells

MDPI

**Supplementary Materials:** The following supporting information can be downloaded at www.mdpi.com/xxx/s1, Figures S1-S8; Tables S1-S3.

Figure S1: Vulnerability of CFTR-KD epithelium to PAO1 strains with attenuated virulence at 6h and 16h post-infection

Figure S2: mRNA expression level of junctional proteins in CFTR-CTL and CFTR-KD epithelia

Figure S3: Expression of junctional proteins in CFTR-CTL and CFTR-KD monolayers

Figure S4: Restoration of ZO-1 expression and localization in apically rehydrated CFTR-KD-epithelium

Figure S5: Protection of CFTR-KD epithelium to Δ*fliC* and Δ*lasR* strains by apical rehydration

Figure S6: Protection of CFTR-KD epithelium to Δ*fliC* strain by previous apical rehydration

Figure S7: Contribution of the secretome to PAO1 growth

Figure S8: Immune gene profile of CFTR-CTL and CFTR-KD epithelia subjected to apical liquid manipulations

Table S1: List of products

Table S2: List of primer pairs used for qPCR

Table S3: List of immunology genes evaluated in uninfected and Δ*lasR*-infected CFTR-CTL and CFTR-KD epithelia after apical liquid manipulations

**Author Contributions:** Conceptualization of the study, M.C. and J.L.S.; conceptualization of the infection experiments, A.L., D.L. and T.K..; methodology, investigation and formal analysis for infection experiments, J.L.S., A.L. and D.L.; methodology, investigation and formal analysis for all other experiments, J.L.S.; investigation for experiments on epithelial cells, M.B.; formal analysis, D.L., C.v.D. and T.K.; writing – original draft preparation, J.L.S. and M.C.; supervision, project administration and funding acquisition, M.C.. All authors reviewed the manuscript.

**Funding:** This research was funded by the Swiss National Science Foundation, grant number 310030_204167/1.

**Institutional Review Board Statement:** "Not applicable" for studies not involving humans or animals.

**Data Availability Statement:** The dataset for this article can be found at the following DOI: 10.26037/yareta:ckvglc7mgbdrjml5k6nllgtlhe. It will be preserved for 10 years.

**Acknowledgments:** We are grateful to Marc Bacchetta for his technical contribution. We would also like to thank the Faculty of Medicine University of Geneva Core Facilities (BioImaging and iGE3) for providing excellent technical support.

**Conflicts of Interest:** The authors declare no conflict of interest. The funders had no role in the design of the study; in the collection, analyses, or interpretation of data; in the writing of the manuscript, or in the decision to publish the results.

## Appendix

Supplementary data

**Figure S1.** Vulnerability of CFTR-KD epithelium to PAO1 strains with attenuated virulence at 6h and 16h post-infection

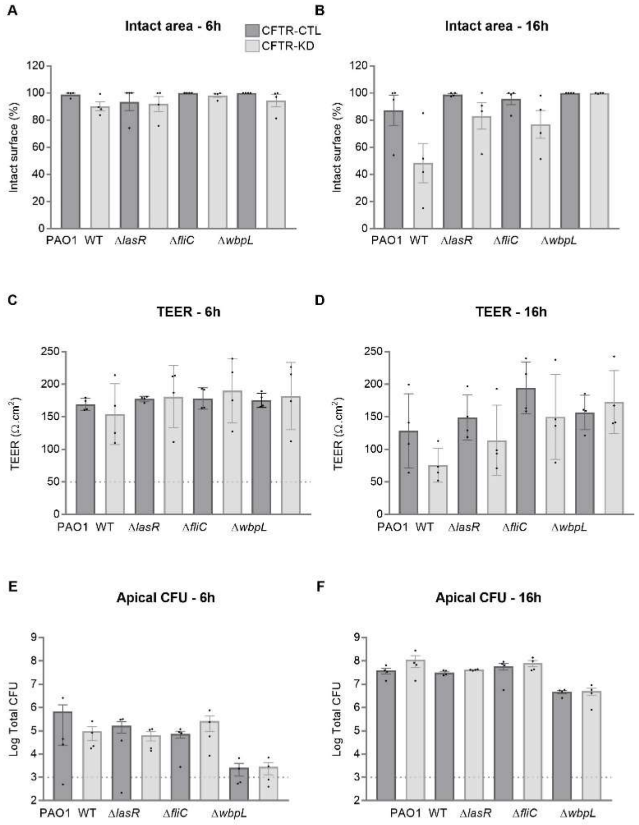


**Figure S1.** CFTR-CTL (dark gray) and CFTR-KD (light gray) epithelium were apically infected with $10^3$ CFU of the wild type PAO1 strain PAO1 mutants (Δ*lasR*, Δ*fliC*, Δ*wbpL*) for 6h (**A**, **C**, **E**) and 16h (**B**, **D**, **F**). The intact epithelial surface (**A**, **B**), TEER value (**C**, **D**) and apical CFUs (**E**, **F**) were determined. (**A**, **B**) The intact epithelial surface was expressed as % of the initial area measured in uninfected conditions (6h: $N$=4, $n$≥1; 16h: $N$=4, $n$=1). (**C**, **D**) TEER values, which were measured in duplicate for each insert, were expressed in $\Omega.cm^2$. The dotted line indicates the average TEER values of empty Transwell filters (6h: $N$=4, $n$≥2; 16h: $N$=4, $n$=2). (**D**, **E**) The dotted line represents the initial amount of inoculated bacteria (6h: $N$=4, $n$≥2; 16h: $N$=4, $n$=2). * indicates the degree of significance, two-way Anova tests.

**Figure S2.** mRNA expression level of junctional proteins in CFTR-CTL and CFTR-KD epithelia

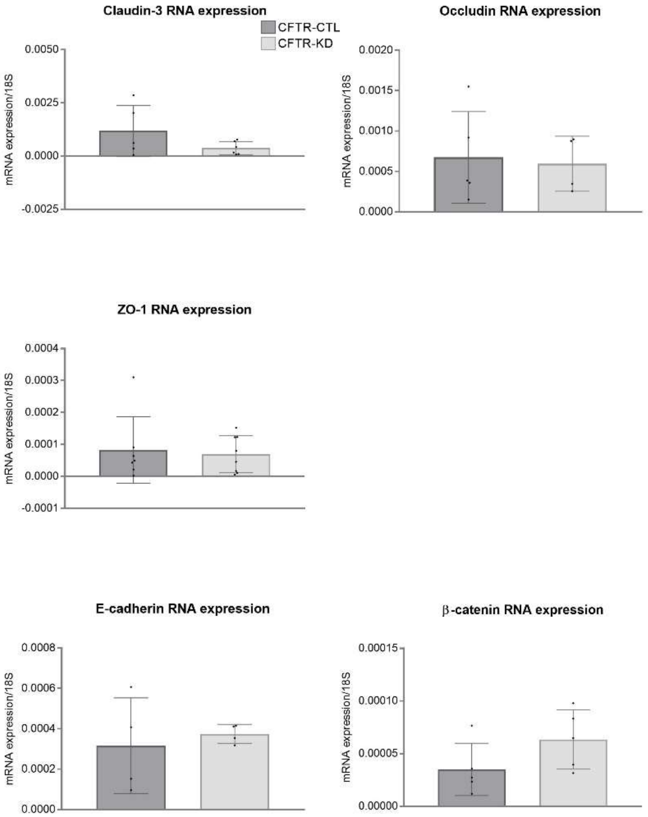


**Figure S2.** Quantification of claudin-3 (*N*≥5, *n*=3), occludin (*N*≥4, *n*=3), ZO-1 (*N*≥7, *n*=3), Ecadherin (*N*=4, *n*=3) and β-catenin (*N*=5, *n*=3) mRNA level expression in CFTR-CTL (dark gray) and CFTR-KD (light gray) epithelia relative to 18S control RNA level. No difference was detected between CFTR-CTL and CFTR-KD filters, paired Student tests.

**Figure S3.** Expression of junctional proteins in CFTR-CTL and CFTR-KD monolayers.

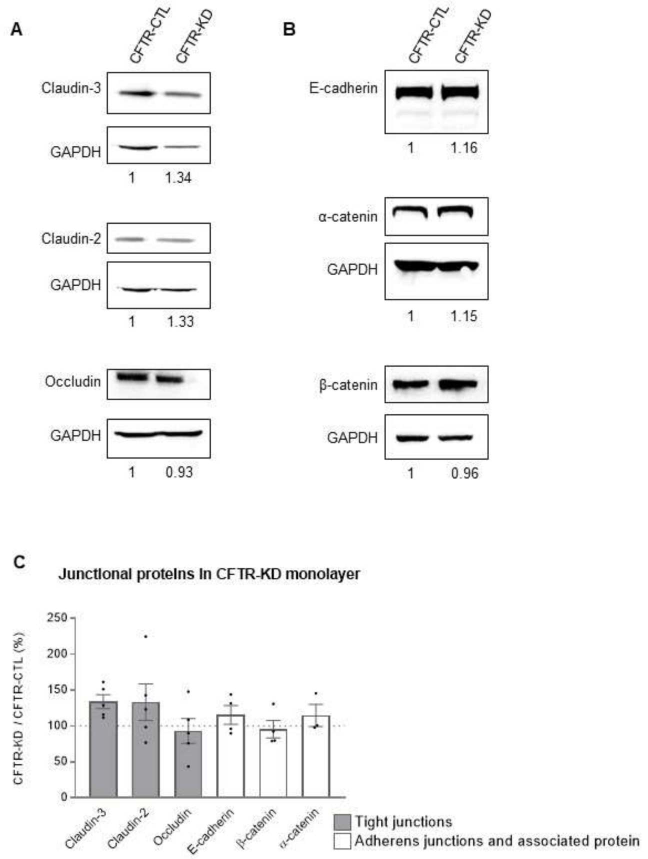


**Figure S3.** Representative western blots for tight junction (**A**) and adherens junction (**B**) proteins. The relative expression level is indicated below each lane. (**C**) Quantification of claudin-3 ($N$=5, $n$=1), claudin-2 ($N$=5, $n$=1), occludin ($N$=5, $n$=1), E-cadherin ($N$=4, $n$=1), β-catenin ($N$=4, $n$=1) and $\alpha$-catenin ($N$=3, $n$=1) expression in CFTR-KD epithelia relative to their expression in CFTR-CTL cells (dotted line). No difference was detected between CFTR-CTL and CFTR-KD monolayers, paired Student tests.

**Figure S4.** Restoration of ZO-1 expression and localization in apically rehydrated CFTR-KD-epithelium

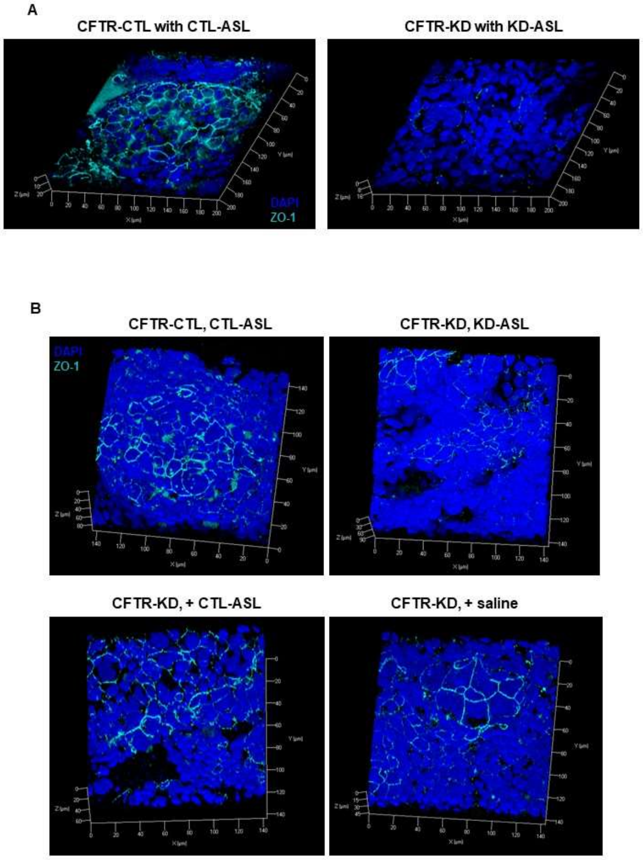


**Figure S4.** ZO-1 expression and localization were evaluated by immunofluorescence. (**A**) Representative images from z-stacks confocal acquisitions of immunofluorescence for ZO-1 detection on CFTR-CTL (left panel) and CFTR-KD (right panel) Transwell inserts (*N*=8). DAPI: dark blue staining; ZO-1: light blue staining. (**B**) Representative images from z-stacks confocal acquisitions of CFTR-CTL and CFTR-KD epithelial immunostained for ZO-1. Apical transfer of CTL-ASL or saline for 24-48h restored the cell-cell expression of ZO-1 in CFTR-KD cultures (*N*=3). DAPI: dark blue staining; ZO-1: light blue staining.

**Figure S5.** Protection of CFTR-KD epithelium to Δ*fliC* and Δ*lasR* strains by apical rehydration

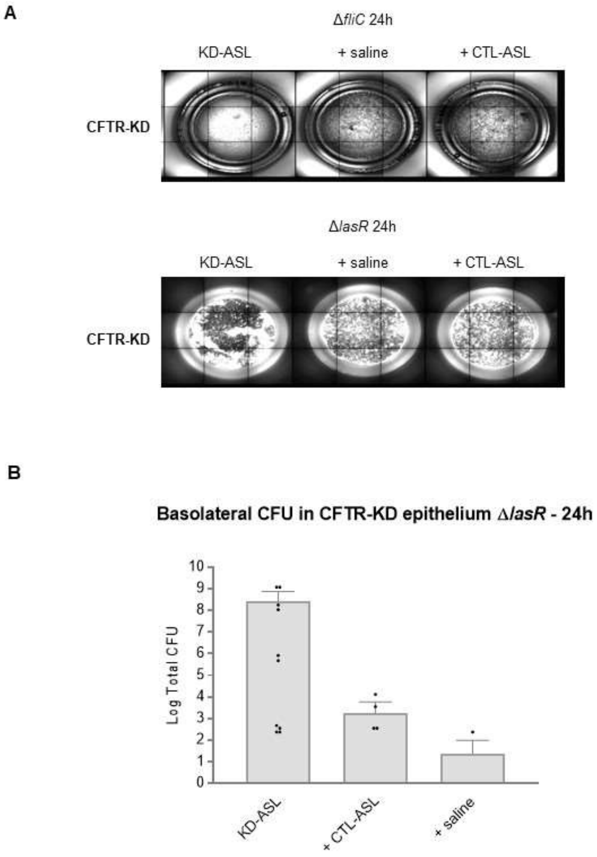


**Figure S5.** (**A**) Representative images from CFTR-KD epithelium infected with Δ*fliC* and Δ*lasR* for 24h. (**B**) Basolateral CFU (*N*=6, *n*≥1) determined after infection with *ΔlasR* for 24h before and after ASL rehydration with CTL-ASL or saline. Points with a value of zero do not appear in logarithmic scale.

**Figure S6.** Protection of CFTR-KD epithelium to Δ*fliC* strain by previous apical rehydration

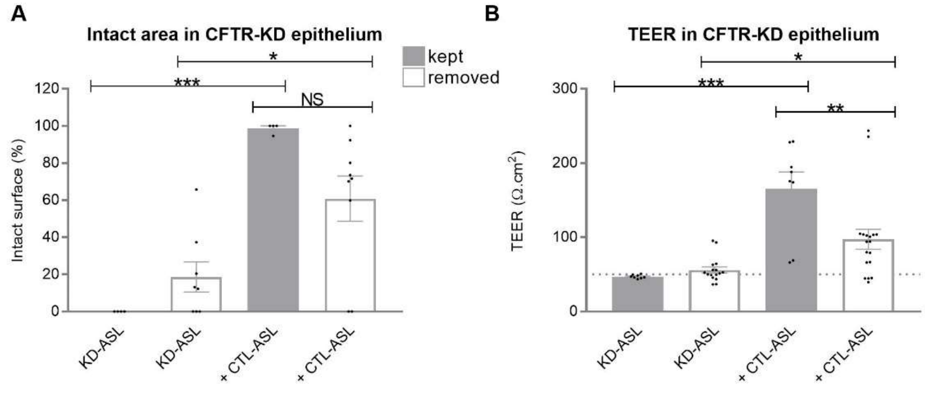


**Figure S6.** The KD-ASL were rehydrated with CTL-ASL for 48h, which were kept (filled bars) or removed (open bars) from the apical surface before infection with $10^3$ CFU of Δ*fliC* for 24h. The intact epithelial surface (**A**) and TEER values were determined. (**A**) The intact epithelial surface was expressed as % of the initial area measured in uninfected conditions (kept: *N*=4, *n*=1; removed: *N*=5, *n*≥1). (**B**) TEER values (Ω.cm²) were measured in duplicate for each insert. The dotted line indicates the average TEER values of empty Transwell filters (kept: *N*=4, *n*=2; removed: *N*=5, *n*≥2). *, **, *** indicate the degree of significance, two-way Anova tests.

**Figure S7.** Contribution of the secretome to PAO1 growth

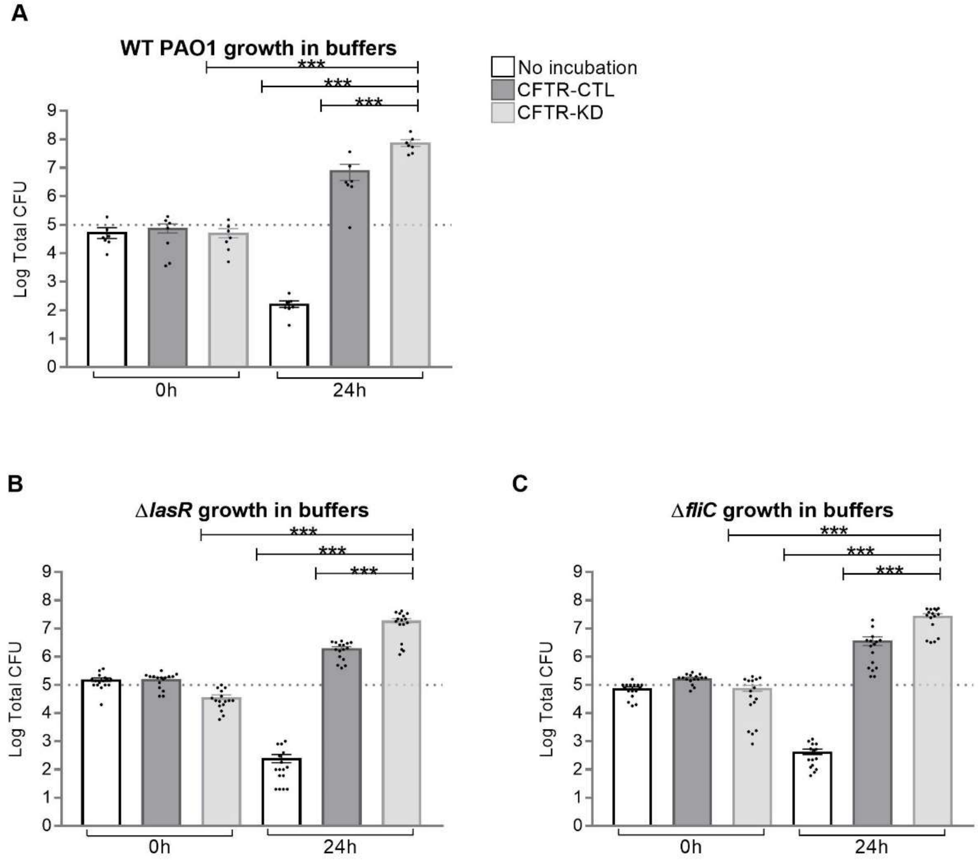


**Figure S7.** ASLs were removed from CFTR-CTL and CFTR-KD cultures and replaced with 95μL of physiological saline for 4h. The conditioned salines with secretomes from CFTR-CTL (dark gray) and CFTR-KD (light gray) cultures were collected and infected with $10^5$ CFU of WT PAO1 ($N$=7, $n$≥2) (**A**), Δ*lasR* ($N$=4, $n$=4) (**B**) or Δ*fliC* ($N$=4, $n$=4) (**C**) for 24h. CFU were counted in conditioned and in control saline at time 0 and 24h after infection. The dotted lines in **A**, **B** and **C** represent the initial amount of inoculated bacteria. *** indicate the degree of significance, two-way Anova tests.

**Figure S8.** Immune gene profile of CFTR-CTL and CFTR-KD epithelia subjected to apical liquid manipulations

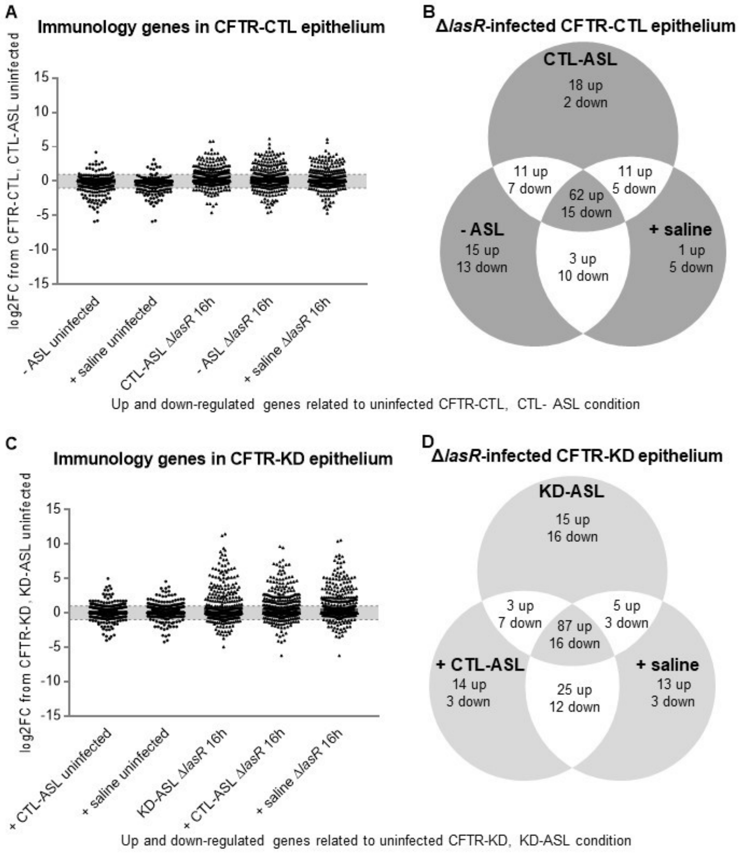


**Figure S8.** (**A**) Dot plots showing distribution of immune gene expression in CFTR-CTL cultures after removal of the CTL-ASL, replacement of CTL-ASL with physiological saline, infection for 16h with $10^3$ CFU of Δ*lasR*, infection with Δ*lasR* in the absence of CTL-ASL or infection with Δ*lasR* while CTL-ASL was replaced with physiological saline. (**B**) Venn diagrams showing distribution of uniquely and commonly upregulated (blank) and downregulated (dark gray) gene counts in Δ*lasR*-infected CFTR-CTL cultures subjected to ASL manipulation. (**C**) Dot plots showing distribution of immune gene expression in CFTR-KD cultures after addition of the CTL-ASL, addition of physiological saline, infection for 16h with $10^3$ CFU of Δ*lasR*, infection with Δ*lasR* while CTL-ASL was added to the KD-ASL or infection with Δ*lasR* while saline was added to the KD-ASL. (**D**) Venn diagrams showing distribution of uniquely and commonly upregulated (blank) and downregulated (light gray) gene counts in Δ*lasR*-infected CFTR-KD cultures subjected to ASL manipulation. Gene expression was normalized to the initial uninfected CFTR-CTL (**A**, **B**) or CFTR-KD (**C**, **D**) conditions. A two-fold change was considered significant.

**Table S1**: List of products

| **REAGENT or RESOURCE** | **SOURCE** | **IDENTIFIER** |
|---|---|---|
| Calu-3 cell line | ATCC | *HTB-55™* |
| | **Antibodies** | |
| Claudin-3 | Abcam | ab15102 |
| Occludin | Invitrogen | 71-1500 |
| Claudin-2 | Abcam | ab53032 |
| GAPDH | Merck | MAB374 |
| β-actin | Sigma | A1978 |
| α1-catenin | Abcam | ab51032 |
| ZO-1 | Invitrogen | 61-7300 |
| β-catenin | Cell Signaling | 8480S |
| E-cadherin | Cell Signaling | 3195S |
| Goat anti-Rabbit HRP | Sigma | Cat# A8275, RRID:AB_258382 |
| Goat anti-Mouse HRP | Sigma | Cat# A5278, RRID:AB_258232 |
| Alexa Fluor 647 goat anti-rabbit (H+L) | ThermoFisher | Cat# A-21245, RRID:AB_2535813 |
| | **Other reagents** | |
| MEM *GlutaMAX* | Gibco® | 41090-28 |
| Fetal Bovine Serum (FBS) | Gibco® | 10270-106 |
| *Non-essential amino acids 100X* | Gibco® | 11140-035 |
| *HEPES 1M* | Gibco® | 15630-056 |
| *Sodium pyruvate 100X* | Gibco® | 11360-039 |
| Penicillin/Streptomycin/Fungizone® | BioConcept | 4-02F00-H |
| Nonidet-P40 | AppliChem | A1694 |
| complete™ *Protease Inhibitor* Cocktail | Roche | 04693124001 |
| Pierce BCA protein assay kit | ThermoFisher | 23228 |
| SDS-PAGE | Bio-Rad | 161-0301 |
| Porablot NCP nitrocellulose membrane | Macherey-Nagel | 741280 |
| BSA | Sigma | A7906 |
| *PBS* | Gibco® | 14190-094 |
| Tween | PanReac Applichem | A4974 |
| HRP substrate Immobilon™ Western | Millipore | WBKLS0500 |
| Trypsin-EDTA 10X | Gibco® | 15400-054 |
| Paraformaldehyde (PFA) | Sigma | 158127 |
| Triton 100X | Sigma | T8787 |
| DAPI | AppliChem | A4099 |
| *Amiloride* | Millipore | 129876 |
| *Forskolin* | Sigma | F6886 |
| *IBMX* | Sigma | I5879 |
| GlyH-101 | Merck | 219671 |
| *B*umetanide | Sigma | B3023 |
| MgATP | Sigma | A9187 |
| RNeasy kit | Qiagen | 74106 |
| nCounter inflammation panel Human v2 | NanoString® | XT_PGX_HuV2_Inflammation_CSO |
| QuantiTect Reverse Transcription Kit | Qiagen | 205311 |
| PowerUp™ SYBR™ Green Master Mix | Appliedbiosystems | A2574 |

**Table S2:** List of primer pairs used for qPCR

| Gene | Forward | Reverse |
|---|---|---|
| Claudin-3 | CTGCTCTGCTGCTCGTGTCC | TTAGACGTAGTCCTTGCGGTCGTAG |
| Occludin | CACACAGGACGTGCCTTCAC | GAGTATGCCATGGGACTGTCAA |
| ZO-1 | CAGCCGGTCACGATCTCCT | TCCGGAGACTGCCATTGC |
| E-cadherin | GGTTATTCCTCCCATCAGCT | CTTGGCTGAGGATGGTGTA |
| β-catenin | TCGCCAGGATGATCCCAGC | GCCCATCCATGAGGTCCTG |
| 18S | GTAACCCGTTGAACCCCATT | CCATCCAATCGGTAGTAGCG |
